# Studying lateralization changes in the aging brain

**DOI:** 10.18632/aging.204550

**Published:** 2023-02-21

**Authors:** Sara Magalhães Ferreira, Koen Cuypers, Melina Hehl

**Affiliations:** 1 Neuroplasticity and Movement Control Research Group, Rehabilitation Research Institute (REVAL), Hasselt University, Diepenbeek, Belgium; 2 Movement Control and Neuroplasticity Research Group, Department of Movement Sciences, Group Biomedical Sciences, KU Leuven, Heverlee, Belgium; 3 Leuven Brain Institute (LBI), KU Leuven, Leuven, Belgium

**Keywords:** lateralization, aging, symmetry, TMS, fMRI

Although, at first sight, one might assume that the human brain is constructed in a roughly symmetric fashion, at closer investigation it becomes apparent that it is inherently asymmetric, i.e., that homotopic brain regions show structural and functional differences [1]. This applies on a structural/anatomical level with, e.g., the right frontal lobe and left occipital lobe tending to protrude over the centerline in a counterclockwise manner called the Yakovlevian torque, but also on the level of brain activity, with certain functions being linked to asymmetric areas of brain activity, such as language which is mainly (but not exclusively) located in the left hemisphere. However, it has been demonstrated that with advancing age, this functional asymmetry of the brain undergoes plastic changes [1].

Over the years, several models have been developed to explain age-related changes in brain asymmetry, such as the Hemispheric Asymmetry Reduction in Older Adults (HAROLD), the right-hemisphere aging, and the Scaffolding Theory of Aging and Cognition (STAC) model ([2] for review). Even though each model succeeds in explaining a subset of brain changes, none accomplishes to serve as an all-encompassing explanation. Therefore, another more recent attempt categorizes the existing evidence on brain aging into three main lines of interpretation, also applicable to brain lateralization: dedifferentiation, neural inefficiency, and compensatory plasticity [3, 4]. In brief, the dedifferentiation model assumes an age-related reduction in the signal-to-noise ratio and specialization of brain regions, resulting in an over-recruitment of task-specific and -unspecific brain regions in older versus younger adults; the neural inefficiency model hypothesizes a diminished signal processing efficiency of the aging brain, leading to a compensatory over-recruitment of task-specific brain regions; and the compensatory neural plasticity model describes (like the dedifferentiation model) an increased task-specific and -unspecific over-recruitment of brain regions with advancing age, however not as the result of malfunctioning but rather of compensatory functional reorganization [4].

As previously stated, advancing age simultaneously impacts lateralization structurally and functionally and a multitude of techniques are employed to study these changes. Structural changes in brain lateralization can be examined using neuroimaging. For example, alterations in the ratio between the two hemispheres’ local cortical thickness, gray matter volume (e.g., using voxel-based morphometry analysis) or white matter connectivity (e.g., as assessed with diffusion-weighted imaging) of homologous brain regions can yield information about the brain’s structural aging process [4].

At the intersection of brain structure and function, non-invasive brain stimulation techniques such as transcranial magnetic stimulation (TMS) are powerful tools to examine the cortex’s lateralization. For example, single-pulse (sp)TMS can be used for investigating cortico-spinal excitability (CSE) and the spatial extent and localization of a muscle’s cortical motor representation (i.e., motor map) at the primary motor cortex (M1). In addition, dual-site (ds)TMS can be applied to study the interaction of a motor-related brain region and M1 [5]. This can be done at rest or during a task for investigating the chronometry of CSE or an interaction on a temporal scale of milliseconds. While evidence on the lateralization of the brain’s motor function using TMS is scarce, our recent work indicated no evidence for age-related differences in lateralization, i.e., in the ratio of the two hemispheres’ CSE, motor map size and volume [6]. Lastly, repetitive (r)TMS can temporarily interfere with a brain region’s function, resulting in a measurable change of behavior. For example, repetitive stimulation of Broca’s area on the left hemisphere interferes with speech, while stimulating the anatomical homologue has no language-related effect. This allows us to study the laterality of a broader set of brain functions such as cognitive tasks [1].

When focusing on brain activation, functional magnetic resonance imaging (fMRI) is a prominent tool to capture the fluctuations in the blood-oxygen-level-dependent (BOLD) signal over time during a task or at rest. Studying task-induced hemodynamic changes in specific brain regions helps to infer their function. More specifically, brain regions engaging synchronously in response to stimuli suggest shared functionality and, altogether, form a connection or network. In the absence of stimuli, i.e., at rest, brain activity is translated into spontaneous low-frequency oscillations in the BOLD contrast, with temporally correlated brain regions constituting a resting-state network. Accumulated fMRI evidence points towards an overall reduction in segregation and specialization of functional networks with aging [7]. Concerning lateralization, these age-related alterations are characterized by a decreased cortical activation of a task-specific brain region, and a concomitant recruitment of the contralateral homologous region, culminating in a widespread reduction in functional brain asymmetry during cognitive and motor performance [2], which can be interpreted as either maladaptive or compensatory (dedifferentiation and compensation model, respectively). In addition to fMRI, other techniques also have potential to examine lateralization changes in brain activity. Firstly, functional near-infrared spectroscopy (fNIRS) is an optical superficial neuroimaging technique to estimate cortical hemodynamic activity. Secondly, oxygen-15 positron emission tomography (PET) provides an indirect measure of blood flow to the brain through a radioisotope. And lastly, surface electrode electroencephalography (EEG) and magnetoencephalography (MEG) respectively measure differences in electric potentials or the resulting changes in magnetic fields, representing the brain's rhythmic activity.

As each technique comes with strengths and limitations, its modality in lateralization research of the aging brain must be critically appraised. For example, spTMS, dsTMS, and rTMS are local techniques that are only informative of a specific region’s function, interaction, or part of a network at a defined point in time. Thus, no inferences about whole-brain networks, a prolonged period of time, or non-cortical brain areas can be made, for which neuroimaging is better suited. In contrast, when measuring brain activity with, e.g., fMRI or EEG, the possibilities to draw conclusions about directionality and causality are limited as compared to TMS. It should also be considered that, based on the choice of analysis pipeline, a brain activity dataset might yield different conclusions as the ground truth remains unknown. Furthermore, although spTMS and dsTMS primarily focus on motor-related regions, they still present higher temporal resolution as compared to fMRI. On the contrary, when examining rTMS-induced behavioral changes, temporal resolution is worse than fMRI. Hence, when investigating the evolution of brain lateralization with advancing age, the choice of technique(s) should be carefully considered in function of the research question.

In conclusion, more research is needed to better understand the brain’s aging process, for which the above-mentioned models and techniques can be valuable tools. However, based on the available evidence, it becomes clear that currently no existing model can accurately describe the whole spectrum of age-related changes in lateralization. Therefore, researchers should be mindful about the pitfall to extrapolate certain patterns of alterations that have been shown for a specific interaction between brain region, research technique, and task-context, as current evidence suggests a much more complex association between lateralization and age than any generalization can provide [1, 3, 4]. In addition, a change in brain structure or activation with advancing age could be interpreted as both, compensatory or maladaptive, depending on whether the resulting behavioral performance is maintained or decreased [3]. Lastly, future aging research should further reinforce multimodal research designs. Combining findings obtained using different techniques and exploring their relationship will lead to a more accurate and complete reflection of the underlying aging processes [4, 8].
